# Controllable Preparation of Gold Nanocrystals with Different Porous Structures for SERS Sensing

**DOI:** 10.3390/molecules28052316

**Published:** 2023-03-02

**Authors:** Yazhou Qin, Dewang Fang, Yulun Wu, Yuanzhao Wu, Weixuan Yao

**Affiliations:** Key Laboratory of Drug Prevention and Control Technology of Zhejiang Province, Zhejiang Police College, Hangzhou 310053, China

**Keywords:** porous material, gold nanocrystals, controlled growth, SERS

## Abstract

Porous Au nanocrystals (Au NCs) have been widely used in catalysis, sensing, and biomedicine due to their excellent localized surface plasma resonance effect and a large number of active sites exposed by three-dimensional internal channels. Here, we developed a ligand-induced one-step method for the controllable preparation of mesoporous, microporous, and hierarchical porous Au NCs with internal 3D connecting channels. At 25 °C, using glutathione (GTH) as both a ligand and reducing agent combined with the Au precursor to form GTH–Au(I), and under the action of the reducing agent ascorbic acid, the Au precursor is reduced in situ to form a dandelion-like microporous structure assembled by Au rods. When cetyltrimethylammonium bromide (C_16_TAB) and GTH are used as ligands, mesoporous Au NCs formed. When increasing the reaction temperature to 80 °C, hierarchical porous Au NCs with both microporous and mesoporous structures will be synthesized. We systematically explored the effect of reaction parameters on porous Au NCs and proposed possible reaction mechanisms. Furthermore, we compared the SERS-enhancing effect of Au NCs with three different pore structures. With hierarchical porous Au NCs as the SERS base, the detection limit for rhodamine 6G (R6G) reached 10^−10^ M.

## 1. Introduction

In the past few decades, noble metal nanocrystals (NM–NCs) have been widely used in the fields of catalysis, sensing, biomedicine, and surface-enhanced Raman spectroscopy (SERS) due to their unique morphology and size-dependent surface plasma resonance characteristics [1,2,3,4,5,6,7,8,9,10]. Among them, SERS, as a fast, non-destructive, and highly sensitive detection technology, has attracted great research interest. As we all know, NM–NCs, as active-matrix materials for surface-enhanced Raman detection, usually play a key role in its sensitivity, especially the morphology of NM–NCs have a decisive effect on their localized surface plasma resonance effects. So far, through the unremitting efforts of researchers, NM–NCs with various morphologies have been prepared, including cube [11,12], octahedron [13,14], dodecahedron [15,16], rod-like [17,18,19,20], flower-like [21,22], and porous structures [23]. Research results show that the sharp tips or narrow nano-gap of NCs often have excellent surface-enhanced Raman effects. Therefore, porous NM–NCs have a large surface area to volume ratio, high density built-in hot spots, and optical properties related to porosity, which have attracted the interest of researchers. Among all kinds of porous metal NCs, porous Au NCs have the stability, biocompatibility, excellent SERS properties of Au NCs, as well as the high specific surface area and high hot spot density of porous materials, which have made it a research hotspot in recent years. For example, Ryu et al. prepared nanopore structured gold nanomaterials by regulating gold nucleation and growth. The minimum detection concentration of R6G reached 10^−6^ M by using the gold nanopore structure as the base material [24]. You et al. reported the preparation of a high-performance surfacing-enhanced Raman scattering (SERS) substrate consisting of mesoporous gold films on flexible cellulose nanofiber paper by a polymer micellar electrochemical deposition method, achieving a minimum detectable concentration of 100 fM for R6G [25]. Choi et al. proposed a simple and green method for the synthesis of a mesoporous gold sponge, which achieved a minimum detection concentration of 10^−8^ M for R6G [26]. To this end, many methods have been developed for the synthesis of well-defined porous Au NCs, including galvanic replacement [27,28], dual templating [29], and dealloying methods [30,31]. In addition, various porous Au NCs with different morphologies were prepared, including nanospheres [32], nanocubes [33], nanorods [34], and nanowires [35]. Among them, the soft template using amphiphilic surfactants is the more effective method for preparing porous metal nanomaterials [36,37,38,39]. Compared with porous metal oxides and other precious metals, the synthesis of porous Au NCs by the soft template method is still a challenge. This is because it is difficult to control the reduction and crystallization of Au around the surfactant template, and the high mobility of AuCl_4_^−^, the precursor of Au, enables it to quickly migrate out of the template. As far as we know, only Liu et al. have successfully prepared mesoporous Au NCs by using the special surfactant C_22_H_45_N^+^(CH_3_)_2_-C_3_H_6_-SH(Cl^−^) as soft template [37]. Although various porous Au NCs have been prepared, the controllable preparation of Au NCs with precise and adjustable structures remains a great challenge.

In this work, we use a surfactant C_16_TAB and GTH dual template to prepare porous Au NCs in aqueous solution. Mesoporous, microporous, and hierarchically porous Au NCs with internal 3D connecting channels were successfully prepared. By systematically exploring the effect of reaction parameters on the growth of Au NCs, we propose the growth mechanism of different porous Au NCs. Furthermore, we explored the SERS-enhancing effect of Au NCs with different pore structures. The hierarchically porous Au with the best SERS enhancement effect was used to detect rhodamine 6G; the detection limit for rhodamine 6G reached 10^−10^ M.

## 2. Results

### 2.1. Structural Characterization

**Figure 1:**
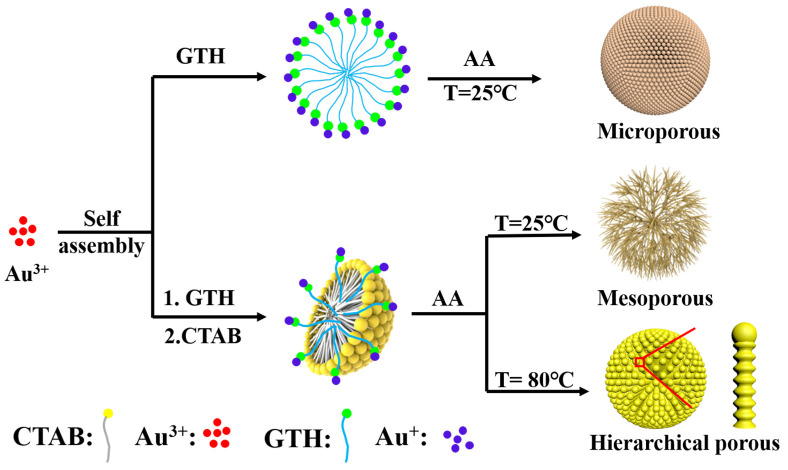
Schematic diagram of the preparation of porous Au NCs.

In this paper, we successfully prepared three kinds of porous Au NCs with different structures, including microporous, mesoporous, and hierarchical porous Au NCs. As shown in Figure 1, under the condition of 25 °C, in the absence of cetyltrimethylammonium bromide, we will prepare a microporous structure assembled by Au rods. The spacing between the gold rods is less than 2 nm, thus forming a microporous structure. When C_16_TAB was added, we prepared mesoporous Au NCs, and further increased the reaction temperature to 80 °C. The Au NCs we prepared were hierarchical porous Au NCs composed of mesoporous and microporous structures. The experimental conditions of Au NCs with each regular morphology are shown in Table 1.

#### 2.1.1. Characterization of Microporous Au NCs

**Figure 2:**
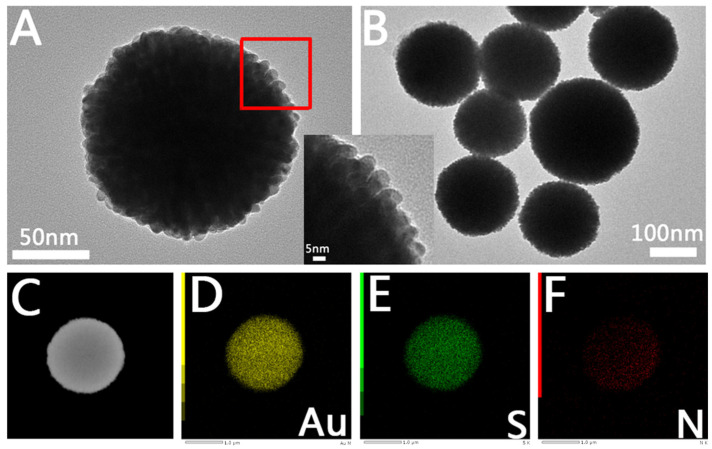
Structural characterization of microporous Au NCs. (**A**) TEM images of single particle microporous Au NCs. The inset is an enlarged view of the part in the red box in (**A**). (**B**) TEM images of low magnification microporous Au NCs. (**C**–**F**) EDS images of microporous Au NCs.

We carried out further structural characterization of Au NCs with different porous structures. The transmission electron microscope (TEM) image of the Au NCs with a microporous structure is shown in Figure 2. Figure 2A is a TEM image of a single microporous Au NC. We can see that the overall morphology is spherical and assembled from Au rods, similar to the dandelion structure. The inset is a magnified view of the part in the red box in Figure 2A; we can see that the size of each Au rod is approximately 7 nm, and the spacing of the Au condition is approximately 0.8 nm. From Figure 2B, we can see that its size ranges from 120–200 nm. Figure 2C–F shows the energy spectrum of Au NCs with a microporous structure. From the figure, we can clearly see that Au NCs with a microporous structure are mainly composed of Au elements, while a small number of S and N elements are distributed on the surface, indicating that GTH is dispersed on the prepared Au NCs.

#### 2.1.2. Characterization of Mesoporous Au NCs

**Figure 3:**
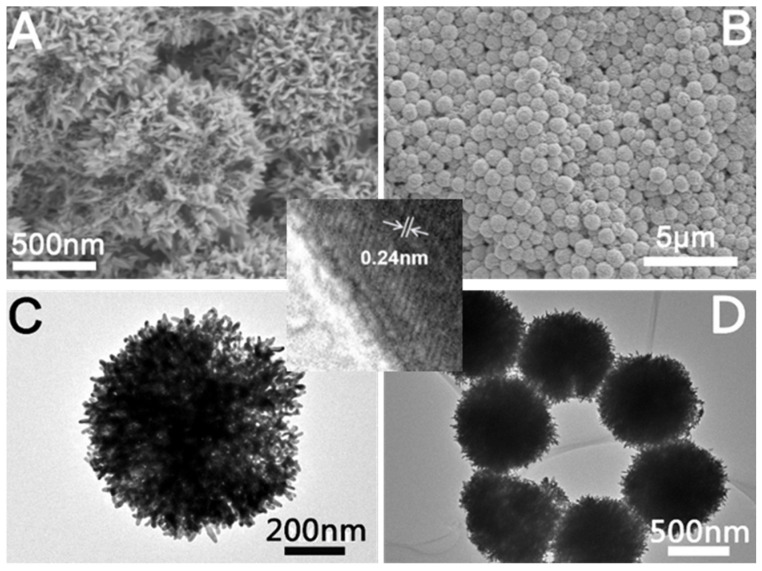
Structural characterization of mesoporous Au NCs. (**A**) High-magnification SEM image, (**B**,**C**) low-magnification SEM image, (**C**) high-magnification TEM image, and (**D**) low-magnification TEM image. The inset is a high-resolution TEM image.

Figure 3A,B shows the SEM images of the prepared mesoporous Au NCs. From Figure 3B, we can see that the prepared mesoporous Au is spherical with a size of approximately 800 nm. From the high-magnification SEM image (Figure 3A), it can be seen that the interior of the sphere is a mesoporous structure. Figure 3C,D are TEM images of the prepared mesoporous Au. From the TEM image of a single mesoporous Au NC (Figure 3C), the mesoporous structure with internal 3D connecting channels can be clearly seen. The HRTEM image insert in Figure 3 was used to further observe the structure of the synthesized Au NCs. As we can see, the lattice spacing is 0.24 nm, which indicates that Au NCs are mainly composed of {111} planes.

#### 2.1.3. Characterization of Hierarchical Porous Au NCs

**Figure 4:**
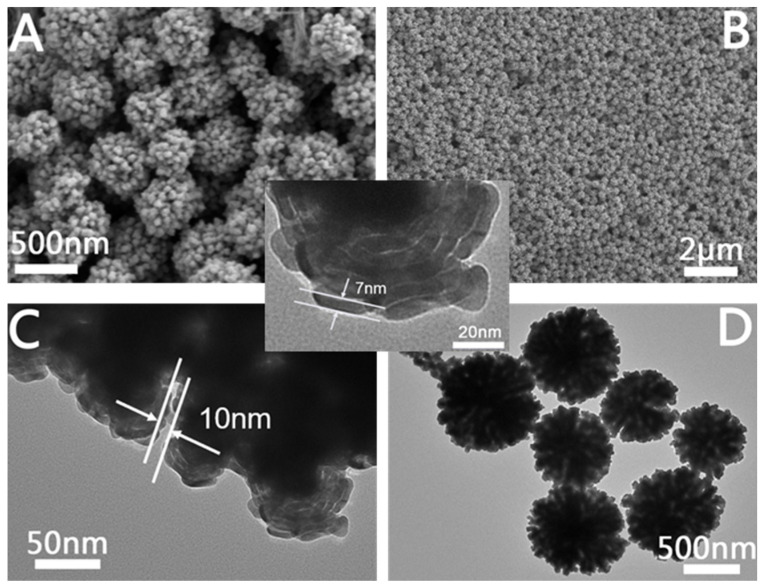
Structural characterization of hierarchical porous Au nanoparticles synthesis at 80 °C. (**A**) High-magnification SEM image, (**B**) low-magnification SEM image, (**C**,**D**) TEM images. The inset is a partial TEM magnification.

Figure 4A,B are SEM images of the prepared hierarchical porous Au NCs at different magnifications. From the SEM image at a higher magnification, we can see that the structure of hierarchical porous Au NCs is a dandelion-like structure. Furthermore, the TEM images of hierarchical porous Au NCs (Figure 4C,D) clearly show that the overall morphology of hierarchical porous Au NCs is spherical. Hierarchical porous Au NCs are assembled from Au nanostrips, and the spacing between the nanostrips is approximately 10 nm. Further, from the local enlarged image of hierarchical porous Au NCs, we can see that the Au nanostrips are assembled from Au wires with a diameter of approximately 7 nm, and the spacing between the Au wires is approximately 0.8 nm. Therefore, hierarchical porous Au NCs not only contain mesoporous structures (10 nm spacing between Au nanostrips), but also microporous structures (0.8 nm spacing between Au wires).

### 2.2. Effects of Reaction Parameters on Au NCs

#### 2.2.1. Effects of GTH on the Au NCs

In order to explore the growth mechanism of porous Au NCs with different structures, we systematically explored the effect of reaction parameters on the Au NCs growth. We first explored the effect of GTH concentrations on Au NCs growth. The TEM image of the porous Au NCs prepared by only changing the amount of added GTH are shown in Figure S1. When only 10 μL GTH was added, the morphology of the prepared Au NCs is shown in Figure S1A. We can see that the Au NCs are spherical structures composed of Au bars, and the Au bars are arranged loosely with a spacing between 2 nm and 50 nm, which is a mesoporous structure. The morphology of Au NCs obtained when the GTH content was increased to 50 μL is shown in Figure S1B. The overall structure is still a spherical structure composed of Au bars but compared with the structure obtained at 10 μL GTH, the Au bars are more closely arranged and the spacing between them is significantly reduced (<2 nm), which is a microporous structure. Further, increasing the amount of GTH to 100 μL will not significantly affect the morphology of the Au NCs, as shown in Figure S1C.

**Figure 5:**
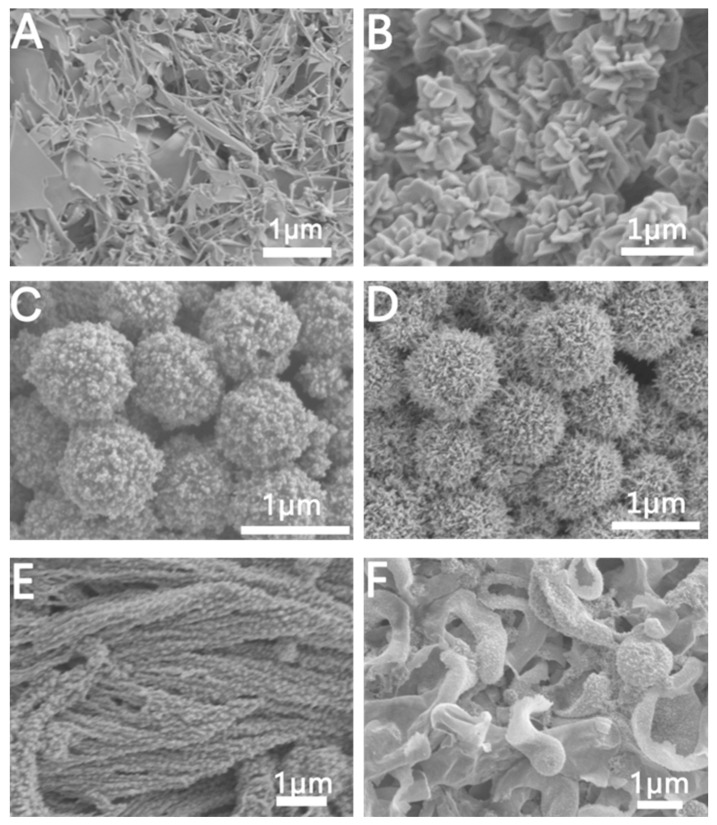
SEM images of Au NCs prepared with different amounts of GTH (2 mM) in the growth solution: (**A**) 0 μL, (**B**) 10 μL, (**C**) 20 μL, (**D**) 50 μL, (**E**) 100 μL, (**F**) 500 μL.

Furthermore, we explored the effect of the amount of GTH on the growth of porous Au NCs in the presence of C_16_TAB. As shown in Figure 5, while keeping other reaction conditions unchanged, we explored its effect by changing the amount of GTH. As shown in Figure 5A, when there is no GTH, we prepared rod-like and sheet-like Au NCs. We infer that in the absence of GTH, the growth of Au is predominantly led by Br^−^ to form rod-like and sheet-like structures, which is consistent with previous reports [40]. When 10 μL of 2 mM GTH was added, we prepared flake-shaped Au NCs, as shown in Figure 5B. When we increase the amount of GTH to 20 μL and 50 μL, we will prepare satellite-like (Figure 5C) and mesoporous Au NCs (Figure 5D). This is due to when the concentration of GTH is high, GTH can not only insert into the micelles formed by C_16_TAB, but also act as a reducing agent to reduce Au^+^ in situ, thus forming spherical mesoporous Au NCs (Figure 5D) [41,42]. Further, when increasing the amount of GTH to 100 μL, GTH will be assembled into strips through intermolecular hydrogen bonds, so the Au^+^ is reduced in situ to form wicker Au NCs (Figure 5E) [43]. When the amount of GTH reaches 500 μL, GTH will form β-sheet-like structures, so Au sheet structures are formed after Au^+^ is reduced in situ (Figure 5F) [44], as shown in Figure 5F.

#### 2.2.2. Effects of Temperature on the Au NCs

**Figure 6:**
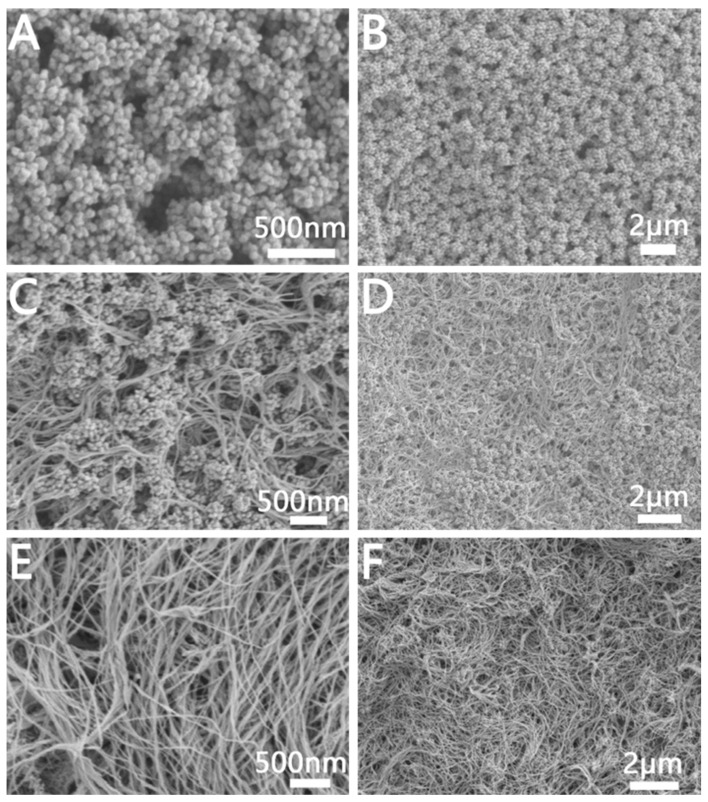
SEM images of Au NCs prepared with different amounts of GSH (T = 80 °C). (**A**,**B**) 50 μL, (**C**,**D**) 150 μL, (**E**,**F**) 250 μL.

The reaction temperature often plays a decisive role in the reaction rate. Therefore, we changed the reaction temperature to explore its influence on the growth of Au NCs. Figure 6 shows the SEM of Au NCs prepared at a reaction temperature of 80 °C. Based on the reaction conditions for the preparation of mesoporous Au NCs, the reaction temperature was adjusted from 25 °C to 80 °C. The SEM images of the Au NCs prepared by us are shown in Figure 6A,B. Its structure is a hierarchical porous structure consistent with the Au NCs shown in Figure 4. When keeping the reaction temperature at 80 °C and increasing the amount of GTH from 50 μL to 150 μL, we will prepare well-divided porous-structured Au NCs and wire-like structures (Figure 6C,D). A further increase in the amount of GTH to 250 μL at a reaction temperature of 80 °C will prepare linear Au, as shown in Figure 6E,F. The diameter of the strips of Au is approximately 30 nm. We reasoned that increasing the reaction temperature not only accelerated the reaction rate but also changed the way GTH was assembled. At lower concentrations of GTH, GTH molecules would be inserted into the micelles formed by C_16_TAB, and intermolecular hydrogen bonds form a folded structure, thus forming a hierarchical porous structure after in situ reduction of Au ions (Figure 6A). With the increase in GTH concentration, more GTH molecules were connected end-to-end through intermolecular hydrogen bonds to form a linear structure, and a thin strip structure was formed after the in-situ reduction of Au ions (Figure 6E).

#### 2.2.3. Effect of C_16_TAB Concentration and Alkyl Chain Length on Au NCs

We further explored the effect of surfactants with different concentrations and alkyl chain lengths on the growth of Au NCs. Figure S2 shows the SEM diagram of the preparation of Au NCs by using hexyl trimethylammonium bromide (C_6_TAB), alkyl trimethylammonium bromide (C_10_TAB), dodecyl trimethylammonium bromide (C_12_TAB), and tetradecyl trimethylammonium bromide (C_14_TAB) instead of C_16_TAB under the condition that other reaction conditions remain unchanged. As shown in Figure S2A, we can see that when the alkyl chain is shorter (C_6_TAB), we get leaf-shaped Au NCs, and cannot get a porous structure. When C_10_TAB is used for alkyl chain growth, we can prepare porous Au NCs, but they are not spherical (Figure S2B). This indicates that when the alkyl chain is short, spherical bilayers cannot be formed and spherical mesoporous Au cannot be obtained. When the alkyl chain is increased to 12 carbons (C_12_TAB), we can get incomplete spherical mesoporous Au NCs (Figure S2C). When the alkyl chain length is increased to 14 carbons (C_14_TAB), we can get complete spherical mesoporous Au NCs (Figure S2D). We further characterized the leaf-shaped Au NCs, as shown in Figure S3A,B with SEM images of leaf-shaped Au NCs. We can see that each Au NC is composed of multiple leaf cross-links. From its TEM image (Figure S3C), we can clearly see the regular leaf-like structure. The HRTEM image (Figure S3D) shows that the lattice spacing is 0.24 nm, which indicates that leaf-shaped Au NCs are mainly composed of {111} planes.

**Figure 7:**
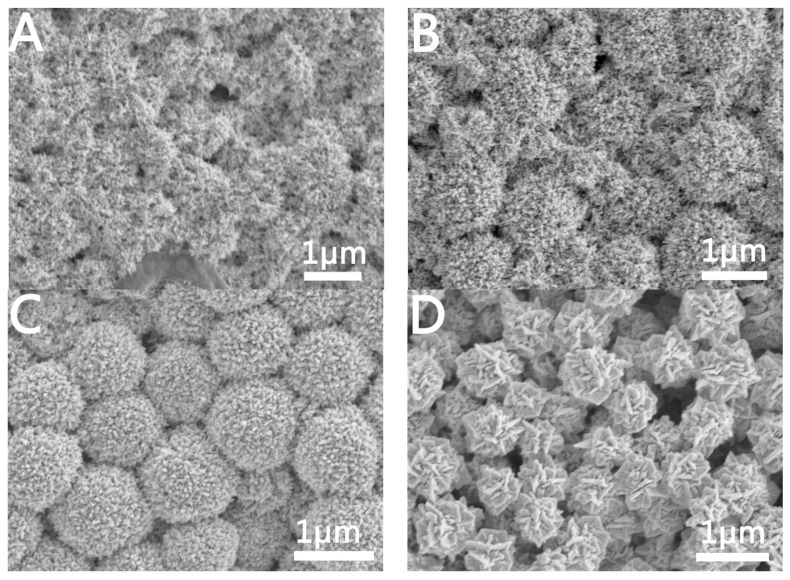
SEM images of gold nanostructures prepared with different amounts of C_16_TAB in the growth solution: (**A**) 5 mg, (**B**) 10 mg, (**C**) 70 mg, (**D**) 120 mg.

Figure 7 shows the SEM images of Au NCs prepared by changing the concentration of C_16_TAB in the reaction at 25 °C. We can see that when only 5 mg of C_16_TAB is added, it will not be enough to form spherical micelles and thus cannot form spherical mesoporous structures (Figure 7A). When the amount of C_16_TAB was increased to 10 mg, the shape of the prepared NCs was a loose spherical mesoporous structure (Figure 7B). In addition, when the amount of C_16_TAB was further increased to 70 mg (Figure 7C), we would obtain Au NCs with a regular mesoporous spherical structure. However, when the amount of C_16_TAB is too large, we will prepare Au NCs with a flower-like structure, forming a large number of platelet-like structures (Figure 7D). We deduce that this is because with the increase of the amount of C_16_TAB, the concentration of Br^−^ in the solution also increases gradually, and Br^−^ will induce the formation of a sheet-like structure, which is consistent with the preparation of sheet-like Au NCs in the presence of only C_16_TAB.

According to the above experimental results, the formation mechanism of porous Au NCs is inferred. While keeping other reaction conditions unchanged, in the absence of C_16_TAB, at 25 °C, GTH in the reaction system will bind to Au^3+^ and reduce Au^3+^ to form polymeric R-S-Au(I) intermediates via covalent bonds, which was consistent with previous reports [37]. Then R-S-Au(I) will self-assemble into spherical micelles to hinder the rapid migration of Au precursors, and under the action of the reducing agent, ascorbic acid, the Au ions are reduced and nucleated in situ along the direction of GTH assembly. When there is less GTH in the reaction system, it will self-assemble into loose micelles and thus form Au NCs assembled by loose Au rods (Figure S1A). When there is more GTH, compact micelles are formed, resulting in the Au NCs forming a tightly assembled microporous structure of Au rods (Figure S1B,C). When C_16_TAB was added to the reaction system, as soon as the precursor AuCl_4_^−^ and C_16_TAB are mixed, the solution color changed from light yellow to brown immediately, indicating the formation of [AuBr_4_]^−^ complexes. Then, the ascorbic acid solution was added, and the solution quickly became colorless, indicating that Au^3+^ was reduced to Au^+^, which is consistent with previous reports. Under conditions higher than the critical micelle concentration of C_16_TAB, it will form spherical micelles. In C_16_TAB micelles, the alkyl chains of C_16_TAB provide nanoscale hydrophobic confined spaces in which hydrophobic molecules can easily diffuse and accumulate to a relatively high concentration. But due to the faster reduction rate and mobility of Au^+^, a spherical mesoporous Au structure cannot be formed only through the C_16_TAB spherical micelles. When a certain amount of GTH is added, the sulfhydryl group in GTH will form stable R-S-Au(I) intermediates with Au^+^ [37,38,39,40,41]. Due to the blocking effect of C_16_TAB, the Au NCs formed by in-situ reduction along the GTH form a large spacing, and thus the mesoporous Au NCs are obtained.

### 2.3. 3 SERS Performance

**Figure 8:**
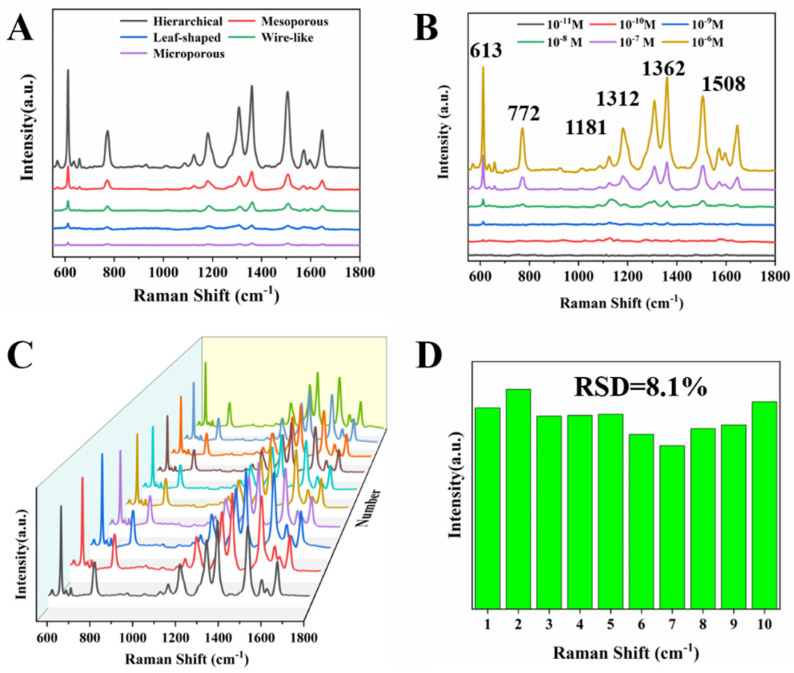
(**A**) Au NCs with different structures were used as SERS substrates to detect 10^−5^ M R6G. (**B**) The hierarchical porous Au NCs were used as SERS substrates to detect different concentrations R6G. (**C**) SERS spectra of 10 different points of R6G. (**D**) Graphs of the intensity of the peaks at 613 cm^−1^ from 10 SERS spectra.

We further take advantage of the large specific surface area and excellent localized surface plasma resonance properties of porous Au NCs for highly sensitive surface-enhanced Raman detection. First, we used 10^−5^ M rhodamine 6G molecule as the analyst to explore the SERS enhancement effect of Au NCs with different morphologies. Figure 8A shows the SERS spectra obtained when Au NCs with different morphologies are used as substrate materials. The SERS characteristic peaks of R6G are located at 613 cm^−1^, 772 cm^−1^, 1181 cm^−1^, and 1312 cm^−1^, which correspond to the C-C-C in-plane bending vibration; out-plane bending vibration of C-H, C-H, and N-H bending vibrations; and C=C stretching vibration, respectively, while the band peaked at 1362 cm^−1^ and 1508 cm^−1^ belonging to the C-C bond and the last one the C=O bond [45,46]. Table S1 shows the attribution of SERS peaks of R6G. Table S2 showed peak intensity of 10^−5^ M R6G at 613 cm^−1^ and 1362 cm^−1^ with different morphology Au NCs as the SERS substrate. We can see that the SERS enhancement effect was hierarchical porous > mesoporous > microporous. We believe that the use of a porous structure for SERS testing should not only consider the contribution of aperture structure to SERS enhancement, but also consider the molecular steric accessibility. When the porous structure had a small pore size to provide excellent SERS enhanced performance and molecular steric accessibility are also satisfied (hierarchical porous), a satisfactory SERS performance could be obtained. Furthermore, we evaluated the SERS properties of hierarchical porous Au NCs, and selected commonly used R6G as the substance to be tested. As can be seen from Figure 8B, under the same test conditions, with the decrease of R6G concentration, the SERS signal gradually decreased, and the lowest detection concentration of R6G molecules reached 10^−10^ M. We further investigated the stability of the SERS test. Figure 8C shows the SERS spectrum of R6G obtained from 10 randomly selected locations using hierarchically porous Au NCs as the base material and 10^−5^ M R6G molecules as the test object. The relative standard deviation (RSD) of the absolute intensity of the peak at 613 cm^−1^ is calculated as 8.1% (Figure 8D), indicating good reproducibility.

## 3. Materials and Methods

### 3.1. Materials

Precursor chloroauric acid trihydrate (99.9% trace metals basis), ascorbic acid (AR, >99.0%), glutathione (Mw = 307, ≥98%), cetyltrimethylammonium bromide (C_16_TAB, AR, 99%), dodecyltrimethylammonium bromide (C_12_TAB, AR, 99%), decyltrimethylammonium bromide (C_10_TAB, AR, 99%), hexyltrimethylammonium bromide (C_6_TAB, AR, 99%), rhodamine 6G (R6G, AR, 99%), nitric acid (HNO_3_, AR), and hydrochloric acid (HCl, AR) were all purchased from Aladdin. The chemical reagents used in the experiments were used directly after purchase without further purification. All the water used in the experiment was ultrapure water (18.2 MΩ·cm) purified through a Milli-Q Lab system (Nihon Millipore Ltd., Merck, Germany). The glassware used in the experiment was washed with the newly made aqua regia (HCl: HNO_3_ = 3:1) for 30 min and rinsed twice with water and ethanol before use.

### 3.2. Instruments

The porous Au NC was characterized by scanning electron microscopy (SEM, JEOL JSM-6700F, Tokyo, Japan), which was carried out with a voltage of 3.0 kV. Transmission electron microscopy (TEM) was operated by Hitachi HT7700 at 100 kV. High resolution TEM (HRTEM) and selected area electron diffraction (SAED) were operated by JEOL JEM-2100F at 200 kV. The surface-enhanced Raman spectroscopy was performed using a Raman spectrometer (Thermo Fisher DXR2xi, Waltham, MA, USA) with laser excitation at 633 nm.

### 3.3. Synthesis of Microporous Au NCs

Firstly, 200 μL 10 mM HAuCl_4_·3H_2_O solution was added to a 20 mL glass bottle containing 5 mL of water, which was stirred for 5 min (300 rpm) at 25 °C. Then, GTH (50 μL, 2 mM) and ascorbic acid (475 μL, 100 mM) were added, which was stirred for 2 min and left to stand for 2 h until the reaction was completed. The products were collected by centrifugation at 8000 rpm, and the collected products were washed twice with water and ethanol, respectively.

### 3.4. Synthesis of Mesoporous Au NCs

Firstly, 200 μL 10 mM HAuCl_4_·3H_2_O solution was added to a 20 mL glass bottle containing 5 mL of water, followed by 30 mg cetyltrimethylammonium bromide (C_16_TAB), which underwent magnetic stirring for 10 min. Then, 0.1 M ascorbic acid (AA) solution of 475 μL was added to the above solution. The color of the solution changes from yellow to colorless, indicating that Au^3+^ is reduced to Au^+^. Then, 50 μL 2 mM GTH solution was added and reacted at 25 °C for 2 h. At the end of the reaction, the products were collected by centrifugation at 8000 rpm, and the collected products were washed twice with water and ethanol, respectively.

### 3.5. Synthesis of Hierarchical Porous Au NCs

The method of preparing hierarchical porous Au nanoparticles is basically the same as that of mesoporous Au, except that the reaction temperature is changed to 80 °C for the above reaction. In order to explore the effects of GTH and C_16_TAB on the growth of Au nanoparticles, we adjusted the amount of GTH and C_16_TAB separately to explore their effects while keeping other reaction conditions unchanged.

### 3.6. SERS Measurements in Colloids

The prepared 10 μL Au nanoparticles were dropped onto the cleaned glass slides, and then 10 μL R6G solutions of different concentrations were added. The solution was pumped and mixed evenly and dried at room temperature to prepare the surface-enhanced Raman spectroscopic detection substrate. A laser Raman spectrometer (Thermo Fisher DXR2xi) equipped with microscope and CCD detector was used to record SERS spectra. The laser wavelength used in the experiment is 633 nm, the acquisition time is 10 s, and the laser power is 6.0 mW. The objective lens (OLYMPUS 50X 0.75 BD) magnification is 50 times. All the collected spectra are baselines corrected by OMNIC for Dispersive Raman, a software provided with the equipment, and later used for analysis. The distribution of the Raman Peak is analyzed by using the matching software OMNIC for Dispersive Raman.

## 4. Conclusions

In conclusion, we developed a facile method for the preparation of porous Au NCs with different structures, and successfully fabricated microporous, mesoporous, and hierarchically porous Au NCs. Microporous Au NCs were controllably prepared under the action of a single ligand, glutathione, while mesoporous Au NCs were controllably prepared under the combined action of dual ligands cetyltrimethylammonium bromide and glutathione. At the reaction temperature of 80 °C, we prepared hierarchical porous Au NCs under the action of dual ligands. In addition, we explored the SERS enhancement effect of the prepared porous Au NCs with different structures, and the lowest detection concentration of rhodamine 6G reached 10^−10^ M. More broadly, this approach offers a promising strategy for designing and constructing multifunctional architectures based on porous noble metal nanostructures. Due to its large specific surface area and local surface plasma resonance, porous noble metal nanostructures can be used in various basic research and applications in biomedical, sensing and detection, catalysis, and other fields.

## Figures and Tables

**Table 1:** Preparation conditions of Au NCs with different morphologies.

	HAuCl_4_/10 mM	GTH/2 mM	C_16_TAB/mg	T/°C	AA/0.1 M	Pore Size
Microporous Au NCs	200 μL	50 μL	0	25	475 μL	0.8 nm
Mesoporous Au NCs	200 μL	50 μL	30 mg	25	475 μL	2 nm–50 nm
Leaf-shaped Au NCs	200 μL	50 μL	30 mg (C6TAB)	25	475 μL	None
Hierarchical porous Au NCs	200 μL	50 μL	30 mg	80	475 μL	0.8 nm and 10 nm
Strip structure Au NCs	200 μL	250 μL	30 mg	80	475 μL	None

## Data Availability

The data presented in this study are available on request from the corresponding author.

## Supplementary Materials

The following supporting information can be downloaded at: https://www.mdpi.com/article/10.3390/molecules28052316/s1. Figure S1: Without CTAB, the Au NCs were prepared by changing the amount of glutathione added. (A) 10 μL, (B) 50 μL and (C) 100 μL.; Figure S2: SEM images of gold nanoparticles prepared under different alkyl length conditions. (A) C6TAB, (B) C10TAB, (C) C12TAB, (D) C14TAB.; Figure S3: Structural characterization of leaf-shaped gold nanoparticles. (A) High-magnification SEM image. (B) and (C) low-magnification SEM image and TEM image. (D) High-resolution TEM image.; Table S1: Characteristic vibrations of R6G.; Table S2: Peak intensity of 10^−5^ M R6G at 613 cm^−1^ and 1362 cm^−1^ with different morphology Au NCs as SERS substrate.

## Sample Availability

Samples of the compounds Au nanocrystals are available from the authors.
