# Supplementary material for: Controllable Preparation of Gold Nanocrystals with Different Porous Structures for SERS Sensing
Source: Molecules. 2023 Mar 2;28(5):2316. doi: 10.3390/molecules28052316 (PMC10004769; doi:10.3390/molecules28052316)
Supplement: Supplementary file 1 [file molecules-28-02316-s001.zip › molecules-2253894-supplementary.pdf]

Supplementary Materials

Controllable Preparation of Gold Nanocrystals with Different Porous Structures for SERS Sensing

Yazhou Qin *, Dewang Fang, Yulun Wu, Yuanzhao Wu and Weixuan Yao *

Key Laboratory of Drug Prevention and Control Technology of Zhejiang Province, Zhejiang Police College, Hangzhou 310053, China

* Correspondence: yazhouqin@zju.edu.cn (Y.Q.); yaoweixuan@zjpc.edu.cn (W.Y.)

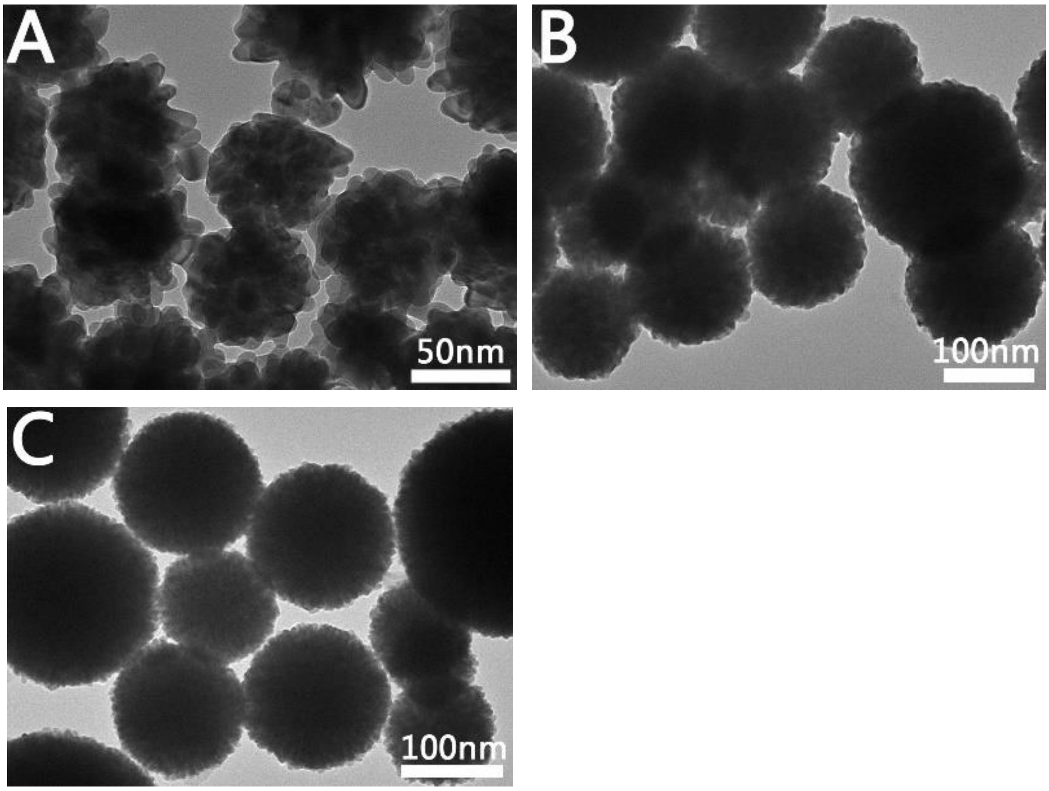

Figure S1. Without CTAB, the Au NCs were prepared by changing the amount of glutathione added. (**A**) 10 μL, (**B**) 50 μL and (**C**) 100 μL.

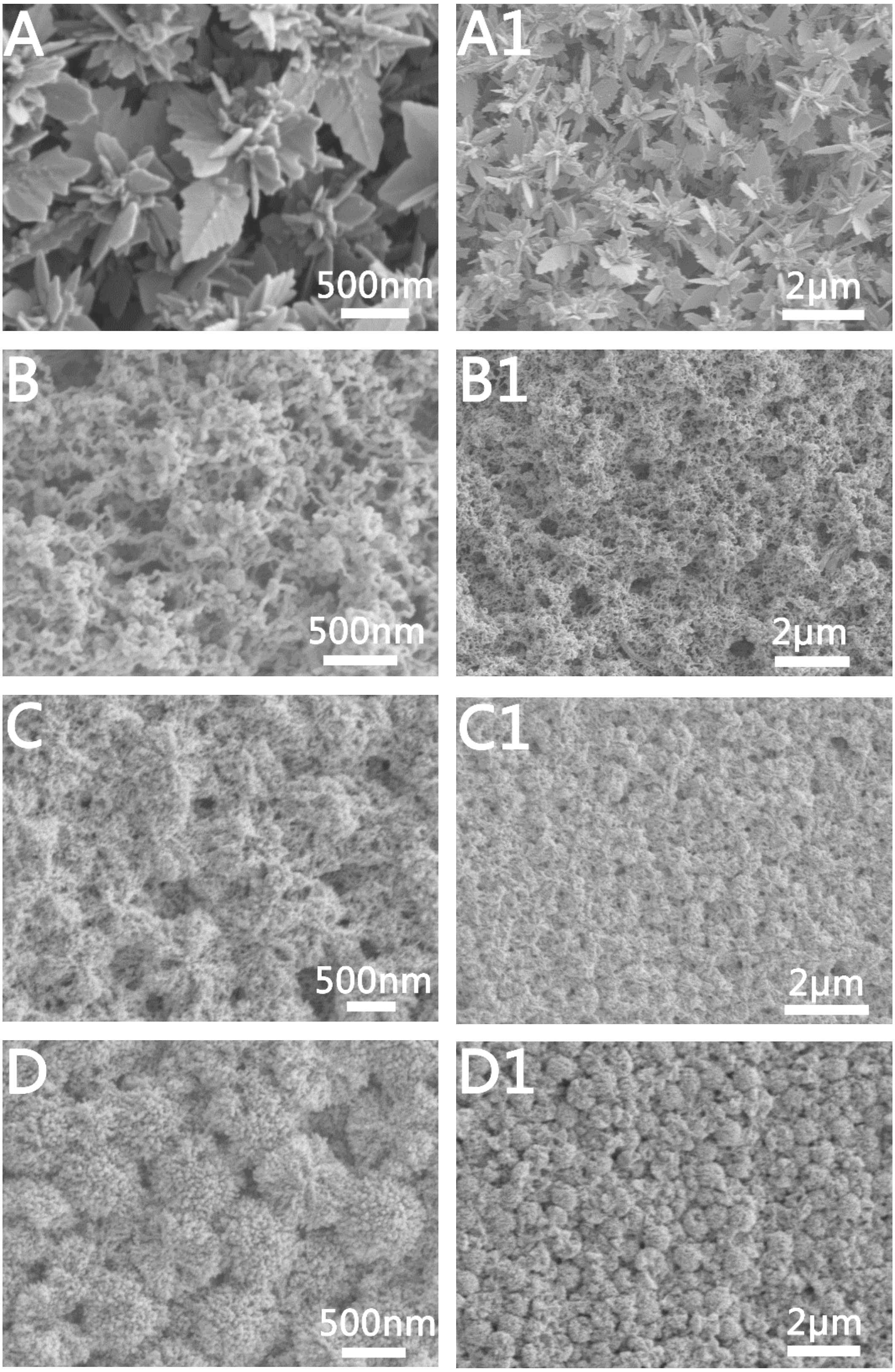

Figure S2. SEM images of gold nanoparticles prepared under different alkyl length conditions. (**A**) C_6TAB, (**B**) $C_{10}TAB$, (**C**) $C_{12}TAB$, (**D**) $C_{14}TAB$.

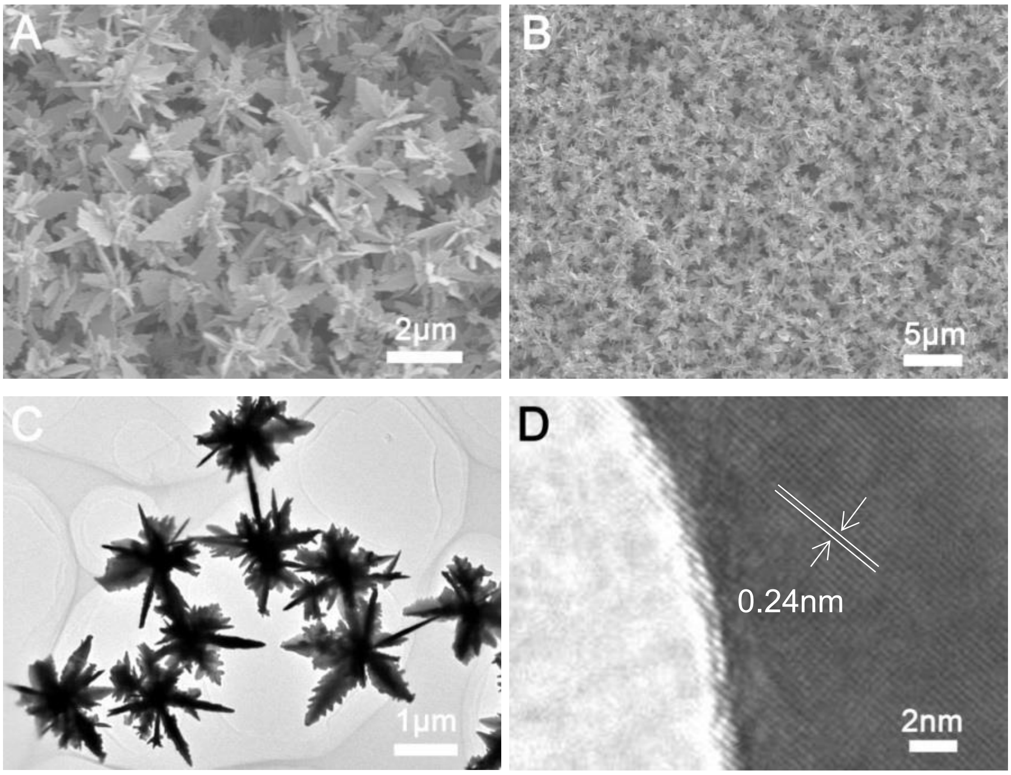

Figure S3. Structural characterization of leaf-shaped gold nanoparticles. (**A**) High-magnification SEM image. (**B**) and (**C**) low-magnification SEM image and TEM image. (**D**) High-resolution TEM image.

Table S1. Characteristic vibrations of R6G.

**SERS/cm^{-1}**	
613	δ(C-C-C)
772	γ(C-H)
1181	δ(N-H), δ(C-H)
1312	ν (C = C)
1362	ν (C = O), ν (C = C)
1508	ν (C = C)benzene, ν (C = O)

Def: ν: stretching; δ: in-plane bending; σ: scissoring; ρ: rocking. γ: out-of-plane bending; τ: twisting; ω: wagging. β: ring breathing.

Table S2. Peak intensity of 10^{-5} M R6G at 613 cm^{-1} and 1362 cm^{-1} with different morphology Au NCs as SERS substrate.

	**613 cm^{-1}**	**1362 cm^{-1}**
Microporous Au NCs	534	413
Leaf-shaped Au NCs	962	887
Strip structure Au NCs	1908	1924
Mesoporous Au NCs	4625	3507
Hierarchical porous Au NCs	19371	16004
